# High-Efficiency Plasmonic Third-Harmonic Generation with Graphene on a Silicon Diffractive Grating in Mid-infrared Region

**DOI:** 10.1186/s11671-018-2750-8

**Published:** 2018-10-25

**Authors:** Junhao Li, Tian Zhang, Lin Chen

**Affiliations:** 1 Wuhan National Laboratory for Optoelectronics, Huazhong University of Science and Technology, Wuhan, 430074 China; 2 State Key Laboratory of Information Photonics and Optical Communications, Beijing University of Posts and Telecommunications, Beijing, 100876 China

**Keywords:** Third-harmonic generation, Graphene, Surface plasmon, Integrated optics

## Abstract

Benefiting from the large third-order nonlinear susceptibility of graphene and significantly enhanced field intensity of graphene plasmons (GPs), graphene has shown great potentials to enhance plasmonic third-harmonic generation conversion efficiency. However, it still lacks an effective configuration that can excite the fundamental frequency (FF) GPs and guide the generated third-harmonic frequency (THF) GPs simultaneously. Here, we have proposed a diffractive silicon grating underneath a graphene sheet to generate and transmit THF GPs. The FF GPs are efficiently excited by illuminating a normal-incidence plane wave due to guided-mode resonance and then are converted to the THF GPs with a large conversion efficiency, originating from the giant field intensity of the FF GPs. We numerically demonstrate that, a large third-harmonic generation conversion efficiency of 3.68 × 10^−7^ can be realized with a small incident power density of 0.19 MW/cm^2^ at 28.62 μm. Furthermore, the generated THF GPs can be efficiently guided along low-loss GP waveguides that are connected to both sides of grating section. Our results can stimulate making graphene-based light sources for mid- and far-infrared silicon photonics.

## Introduction

Harmonic generation is a nonlinear optical process, in which N photons with the same frequency *ω* interacting with a nonlinear material are combined to generate new photons with frequency *Nω*. As a means to extend coherent light sources to short wavelengths, third-harmonic generation (THG) has attracted tremendous research interest. Conventionally, high-efficiency harmonic generation is realized in exotic crystals, but which compromises high-density photonic integration [1]. Silicon has become the mature material choice as an optical information carrier to transmit light signals in highly integrated photonic circuits. Nonlinear optical effects, such as stimulated Raman scattering [2] and THG [3–5], have great potentials to broaden the functionalities of silicon photonics. However, efficient light emission by using silicon remains a challenging issue due to its indirect band gap. Using nonlinear optical interactions, such as THG, seems a rather promising approach to provide coherent light for silicon photonics. In general, the THG conversion efficiency (CE) for an optical waveguide can be enhanced by using phase matching between the fundamental mode and the third-harmonic mode. This method typically requires complicated configurations, which are usually difficult to implement in practical situations. An effective and robust method for enhancing the THG CE can be made by increasing the light intensity within the nonlinear material, which offers us the opportunity to relax the stringent demanding for the phase-matching condition. This has been recently realized by using ultrahigh quality factor slow light silicon photonic crystals [3–5], small-modal-volume silica microrods [6], and surface plasmons [7–10]. It has been reported that silicon photonic crystals have improved the THG CE to the magnitude of ~ 10^−7^ due to the reduced group velocity of c/40 [4]. Quite recently, surface plasmons have been proved capable of increasing the THG CE to the order of 10^−5^ due to the tight electric field enhancement [7].

In recent years, the operation wavelength of silicon photonics has extended to the mid- and far-infrared (IR) regions due to many potential applications such as chemical and biological sensing [11]. The use of plasmonics in mid- and far-IR regions is attractive because the propagation loss of a plasmonic waveguide decreases dramatically at longer wavelengths and also because the mode cross section of such waveguides is subwavelength, which would significantly enhance light-matter interactions such as THG conversion [7–10, 12, 13]. Recent studies have proved that graphene serves as an excellent nonlinear optical material to enhance the nonlinear effect, leading to various applications including four waves mixing [14, 15], THG [16–18], all-optical switching [19], and optical bistability [20, 21], due to its large third-order nonlinear optical susceptibility. Especially, the observed threshold of optical bistability can be greatly reduced, thanks to the large third-order nonlinear optical susceptibility of graphene [20, 21]. More interestingly, in contrast to plasmon mode in metals, graphene plasmons (GPs) have significantly larger wave vectors as well as much higher confinement of light, which indicates the capability of further enhancing the CE of THG [13]. However, a direct coupling between the fundamental frequency (FF) GPs and radiation waves is prevented due to their momentum mismatch, which makes implementing this scheme a difficult issue in practice. It is for this reason that the researchers have employed the guided-mode resonance of gratings to address the coupling issue [12, 18, 20]. The proposed scheme in Ref. [18] is purposely designed to directly excite the FF GPs and hence enhance the CE of third-harmonic frequency (THF) free-space waves in the terahertz domain.

In this article, we have also used the guided-mode resonance of gratings to efficiently excite the FF GPs on the graphene sheets. Different from the configuration in Ref. [18] where the GPs are used to enhance the CE of THF free-space waves in the terahertz domain, here, the GPs are utilized to generate THF GPs at infrared frequencies on a silicon chip. The giant field intensity of FF GPs in combination with large third-order nonlinear susceptibility of graphene results in a noticeably enhanced CE of THF GPs on the graphene sheet in mid- and far-IR regions. We note a previous study on using quasi-phase-matching condition to improve the CE of THF GPs on a graphene surface [13]. However, we emphasize here, although a high CE between FF and THF GP is achievable in Ref. [13], a direct coupling between the radiative waves and GPs is missing. In contrast, the presented scheme not only can directly be coupled with the spatial FF waves, but also can highly efficiently generate the THF GPs, rendering the proposal suitable for integration on a silicon photonic platform. In addition, the demonstrated plasmonic frequency converters have the advantages of compactness and high CE, while requiring a small incident power [22, 23].

## Methods

Graphene’s surface conductivity can be estimated by widely used Kubo formula under the assumption of chemical potential (also termed as Fermi energy), *μ*_*c*_. In the infrared and terahertz frequencies, with |*μ*_*c*_| ≫ *k*_*B*_*T* (*k*_*B*_ is Boltzmann constant, and *T* is the temperature), the surface conductivity of graphene could be approximated as

1 $$ {\displaystyle \begin{array}{l}{\sigma}_g=i\frac{e^2{k}_BT}{\pi {\mathrm{\hslash}}^2\left(\omega +i{\tau}^{-1}\right)}\left[\frac{\mu_c}{k_BT}+2\ln \left(\exp \left(-\frac{\mu_c}{k_BT}\right)+1\right)\right]\\ {}\kern2.25em +i\frac{e^2}{4\pi \mathrm{\hslash}}\ln \left[\frac{2\left|{\mu}_c\right|-\mathrm{\hslash}\left(\omega +i{\tau}^{-1}\right)}{2\left|{\mu}_c\right|+\mathrm{\hslash}\left(\omega +i{\tau}^{-1}\right)}\right]\end{array}} $$

where *e* is the electron charge, ℏ is the reduced Planck’s constant, *ω* is the radian frequency, and *τ* is the momentum relaxation time representing loss mechanism. In our study, the working temperature is assumed to be *T* = 300 K. By taking the individual graphene sheet as a non-interacting monolayer, the optical conductivity of few-layer graphene is *nσ*_*g*_ [24], where *n* is the number of graphene layers (*n* < 6). We model graphene as an anisotropic material and the effective in-plane permittivity can be written as [25, 26].

2 $$ {\varepsilon}_x={\varepsilon}_z=1+\frac{in{\sigma}_g{\eta}_0}{k_0{d}_g} $$

where *η*_0_(=377 Ω) is the impedance of air, *k*_0_ is the wave vector in the air, and *d*_*g*_ is the total thickness of *n*-layer graphene sheets. The out-of-plane permittivity of graphene, *ε*_*y*_, is kept constant at 2.5, regardless of the Fermi level [27, 28].

## Results and Discussion

### Excitation of FF GPs with a Silicon Grating

**Fig. 1:**
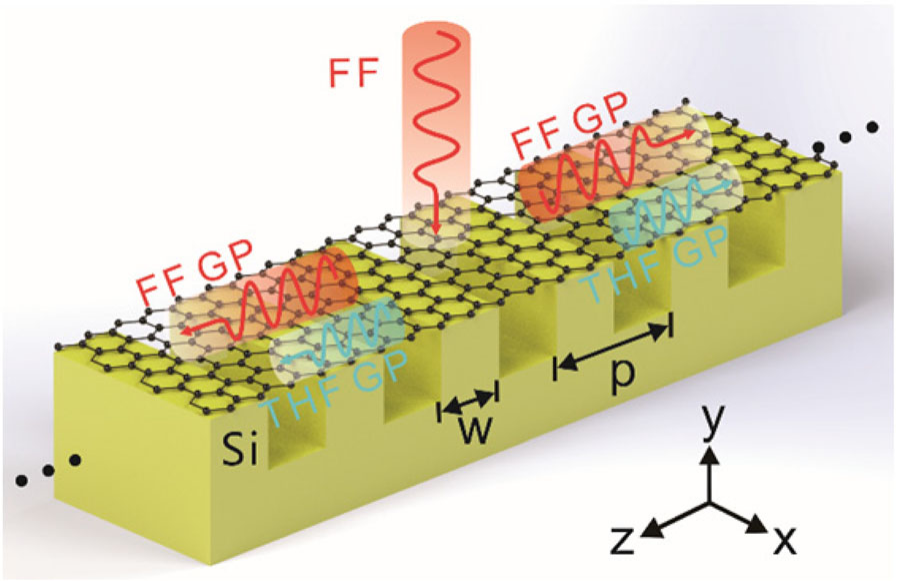
The scheme of the GSSDG as a THG wavelength converter. The FF GPs (red curves) are excited with a normal-incidence x-polarized plane wave of FF and then generate the THF GPs (blue curves) due to the silicon gratings underneath the graphene sheets. The period of the grating is *p*, and *w* denotes the width of silicon

Firstly, we consider the excitation of the FF GPs and the generation of the THF GPs on graphene sheets sustained by dielectric grating (GSSDG) as shown in Fig. 1. Considering the practical situation that the area of graphene can be hundreds of times larger than the grating section, it is assumed that the graphene sheets are flat on the top of gratings and do not conform to the gratings. We have noted some research studies on GP supported by graphene sheets sustained by gratings where the graphene sheets are assumed to flat [12, 13]. Especially, we find the experimental results are well consistent with the simulation results, where the graphene sheets are assumed to be flat in the modeling [12]. The GSSDG is assumed to be infinite along *x* direction and periodic along *z* direction. The thickness of the silicon grating layer underneath the graphene sheets is assumed to be 2 μm. In this case, the grating layer can be considered infinitely thick in the modeling since the silicon substrate below the grating does not affect the field distribution of the GPs in the air-graphene-grating model. The dispersive relationship of the GPs supported by this configuration can be expressed as [29].

3 $$ \frac{\varepsilon_{r1}}{\sqrt{\beta^2-{\varepsilon}_{r1}{k}_0^2}}+\frac{\varepsilon_{r2}}{\sqrt{\beta^2-{\varepsilon}_{r2}{k}_0^2}}=-\frac{in{\sigma}_g}{{\omega \varepsilon}_0} $$

where *β* is the propagation constant of the GPs along *z-*axis, *ε*_0_ is the permittivity in the air, and *ε*_*r*1_(=1) and *ε*_*r*2_ are the dielectric constants of the dielectric mediums above and below the graphene layers, respectively. As the grating period is much smaller than light wavelength in the air, the silicon grating can be approximately modeled as an effective medium with the equivalent permittivity [30].

4 $$ {\varepsilon}_{r2}=f{\varepsilon}_{\mathrm{silicon}}+\left(1-f\right){\varepsilon}_0 $$

where *ε*_silicon_(=11.9) is the permittivity of silicon at infrared and terahertz frequencies [31], and *f*(=*w*/*p*) is the filling ratio of the silicon (*f* is fixed at 0.5 in this work).

**Fig. 2:**
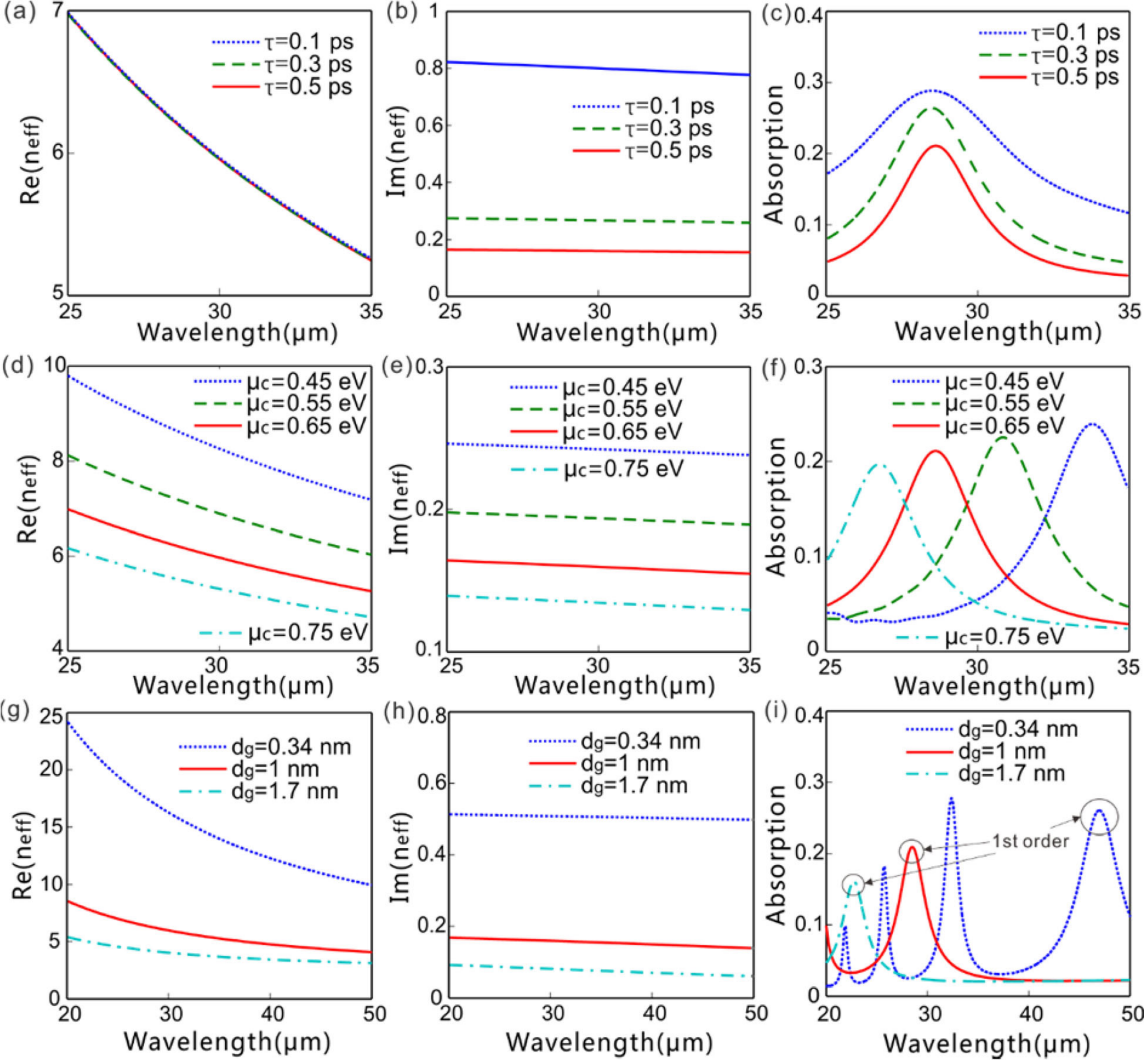
The real [Re(*n*_eff_)] and imaginary [Im(*n*_eff_)] parts of the effective index, and absorption versus wavelength with different values of *μ*_*c*_, *τ*, and *d*_*g*_. **a**–**c** Re(*n*_eff_), Im(*n*_eff_), and absorption versus wavelength (*τ*=0.1, 0.3, and 0.5 ps, associated with *μ* = 0.14, 0.42, 0.69 m^2^V^−1^s^−1^, respectively) with *μ*_*c*_ = 0.65 eV and *d*_*g*_ = 1 nm. **d**–**f** Re(*n*_eff_), Im(*n*_eff_), and absorption versus wavelength (*μ*_*c*_=0.45, 0.55, 0.65, and 0.75 eV) with *τ* = 0.5 ps and *d*_g_ =1 nm. **g**–**i** Re(*n*_eff_), Im(*n*_eff_), and absorption versus wavelength [*d*_*g*_=0.34 nm (*n* = 1), 1 nm (*n* = 3), and 1.7 nm (*n* = 5)] with *μ*_*c*_ = 0.65 eV and *τ* = 0.5 ps. For all the cases, the grating period is fixed at *p* = 4 μm

The dispersion relation of the GPs on the GSSDG for different parameters (*τ*, *μ*_*c*_, and*d*_*g*_) is shown in Fig. 2. In the whole work, two-dimensional finite difference time domain (FDTD) with a commercial software of Lumerical FDTD Solution is conducted to do the numerical modeling. In the simulation of this part, the perfect matched layer boundaries and periodic boundaries are used in the *y* and *z* directions, respectively, while the whole structure is assumed to be infinite along the *x* direction. The mesh sizes with 0.1 nm along *y* direction and 10 nm along *z* direction are used to describe the graphene, while non-uniform meshes with a maximum value of 20 nm along *y* direction and uniform mesh of 10 nm along *z* direction are adopted in the regions besides the graphene sheets. It can be seen from Fig. 2a, d, g that, within the wavelength range considered, the wave vector of the GPs is dozens of times larger than that of the air, which indicates the optical field of the GPs is strongly confined on the graphene surface. However, the phase mismatching between the GPs and the radiation waves prevents the direct coupling between them. The silicon diffractive grating below the graphene sheets shown in Fig. 1 can provide an additional momentum to overcome the wave vector difference so that the FF GPs can be efficiently excited with a plane wave incidence. The grating period, *p*, needs to meet the phase-matching equation as

5 $$ \operatorname{Re}\left({\beta}_{\mathrm{FF}}\right)=j2\pi /p+{k}_0\sin \theta $$

where *β*_FF_ is the propagation constant of the FF GPs along *z*-axis, *j* is the diffraction order, and *θ* is the incident angle. To excite the FF GPs of effective wavelength of *λ*_FF_ with the fundamental diffraction order *j* = 1 under the condition of normal incidence *θ* = 0, the following expression should be satisfied

6 $$ {\lambda}_{\mathrm{FF}}=\operatorname{Re}\left({n}_{\mathrm{eff}}\right)p $$

Figure 2 presents the dependence of the real [Re(*n*_eff_)] and imaginary [Im(*n*_eff_)] part of the effective indices and absorption on light wavelength with different values of *τ*, *μ*_*c*_, and*d*_*g*_. It apparently explains how the parameters of graphene influence the excited FF GPs under the illumination of a normal-incidence x-polarized plane wave of FF, where the grating period is fixed at 4 μm. Both the real [Re(*n*_eff_)] and imaginary parts [Im(*n*_eff_)] of effective refractive indices of the FF GPs decreases with the increase of light wavelength within the considered wavelength range (Fig. 2a, b, d, e, g, h). This means that, with a shorter wavelength of light, GPs are more strongly confined around graphene sheets, resulting in a larger propagation constant and higher propagation loss. The absorption is highly sensitive to wavelength and is sharply increased as the incident wavelength approaches the resonance wavelength (Fig. 2c, f, i). The carrier scattering time *τ* determines the carrier mobility *μ* in graphene as $\tau ={\mu \mu}_c/e{\nu}_F^2$ with the Fermi velocity of *ν*_*F*_ = 9.5 × 10^4^ m/s. Considering that a carrier mobility of *μ* > 10 m^2^V^−1^s^−1^ has been experimentally achieved in high-quality suspended graphene [32], which leads to *τ* > 1.5 ps, our setting of *τ* ≤ 0.5 ps can reflect the practical transport loss of graphene conservatively. The *τ*, associated with the carrier mobility *μ*, gently influences the Re(*n*_eff_) and the excitation wavelength of FF GPs, but greatly affects the Im(*n*_eff_) and absorption (Fig. 2a–c). The enhanced *μ*_*c*_ decreases Re(*n*_eff_) and Im(*n*_eff_) simultaneously, hence reduces the resonance wavelength of FF GPs accordingly (Fig. 2d–f). The Re(*n*_eff_), Im(*n*_eff_), and the resonance wavelength of FF GPs reduce with the increase of the graphene thickness, corresponding to the number of graphene layers (Fig. 2g–i).

**Fig. 3:**
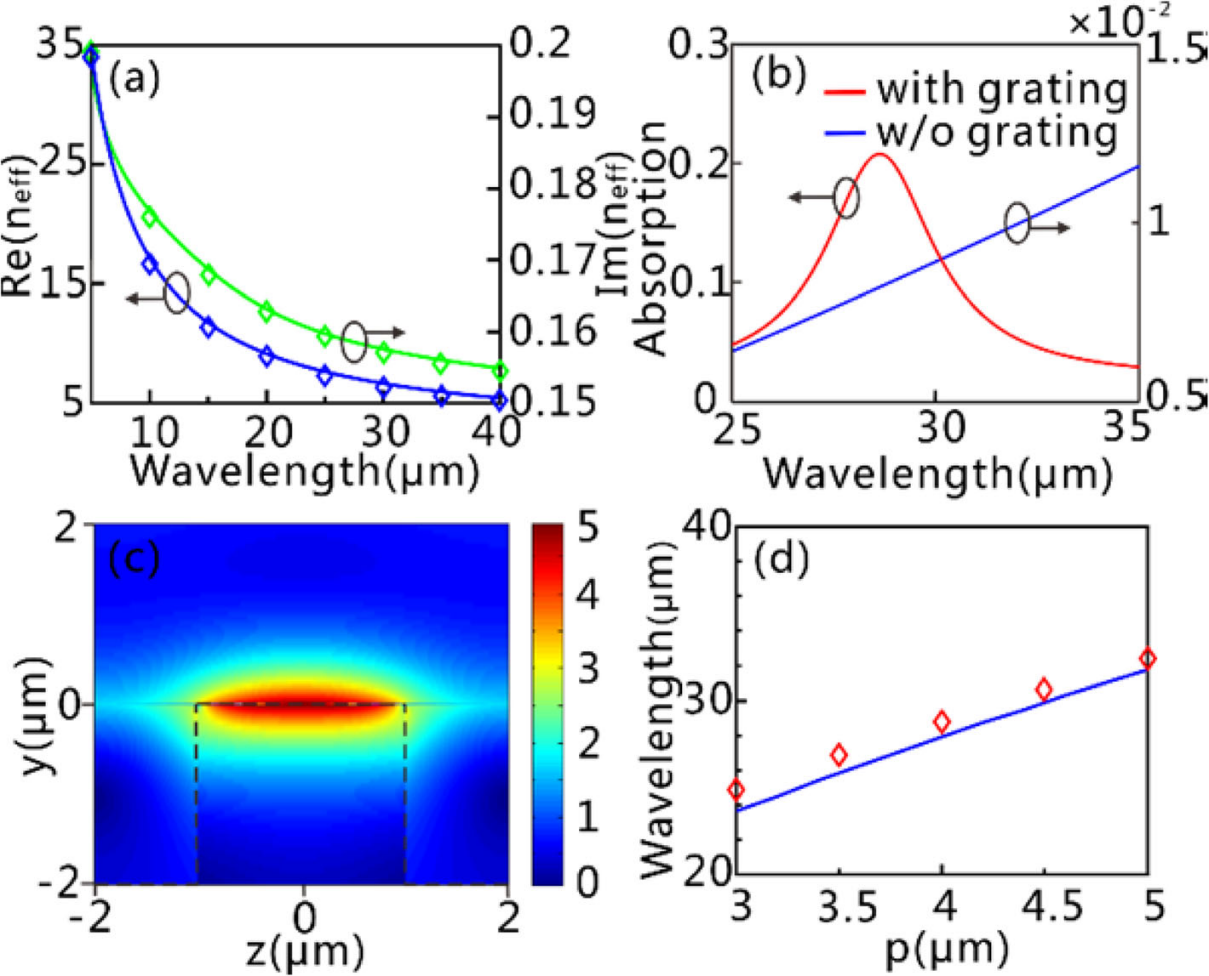
FF GPs and field enhancement on the GSSDG. **a** Dispersion curves of the GPs on the GSSDG. The blue and green solid lines correspond to the real [Re(*n*_eff_)] and imaginary [Im(*n*_eff_)] part of the effective index retrieved from Eq. (3), respectively, while the blue and green rhombuses are gotten from numerical simulations. **b** Absorption spectrums with grating substrate (red line) and pure silicon substrate without the grating (blue line). **c** The normalized |*E*| distributions of the fundamental GPs mode at 28.62 μm. The black dashed lines outline the silicon layer. **d** The excitation wavelength of the fundamental GPs mode versus the grating period. The blue line is retrieved from Eq. (6), and the red rhombuses are from numerical simulation. In **b** and **c**, *P* is set as 4 μm. All the simulation results are retrieved by the commercial software Lumerical FDTD Solutions

In the following, we take *τ* = 0.5 ps, *μ*_*c*_ = 0.65 eV, and *d*_*g*_ = 1 nm as examples. The dispersion relation of the GPs on the GSSDG is shown in Fig. 3a, where the calculated dispersion curves agree well with the simulation results obtained by the commercial software Lumerical FDTD Solutions. Figure 3b shows the optical response of the graphene sheets with and without the silicon grating. It can be clearly seen that the absorption efficiency (over 20%) is significantly enhanced at *λ* = 28.62 μm when the grating is involved (*p* = 4 μm). In contrast, the absorption efficiency is kept at a low level (below 2%) over the entire spectral range considered if the grating is not taken into account. The noticeably enhanced absorption for the former case can be attributed to the excitation of the GPs at *λ* = 28.62 μm. We can find from the |*E*| distributions at *λ* = 28.62 μm (Fig. 3c) that the excited GPs is the fundamental guided-wave resonance mode (*j* = 1). One can see from Fig. 3d that the resonant wavelength of the fundamental mode with respect to the grating period from the numerical simulations, agrees well with the theoretical result predicted by Eq. (6).

It should be noted that, a greatly enhanced plasmonic field on the graphene surface occurs due to the significant reduction of the group velocity of the FF GPs (dozens of times smaller than light velocity in the air). The plasmonic field undergoes electric field enhancement 5 times as high as the illuminating plane waves, which can be highly expected to generate THF GPs with a significantly enhanced CE, in combination with the large third-order optical nonlinearity of graphene [16, 17]. The nonlinear response of graphene can be described by the nonlinear conductivity coefficient defined as [17].

7 $$ {\sigma}_3\left(\omega \right)=i\frac{3{e}^2{\left({ev}_F^2\right)}^2}{32\pi {\mathrm{\hslash}}^2{\mu}_c{\omega}^3} $$

where the Fermi velocity *ν*_*F*_ = 9.5 × 10^4^ m/s.

### Generation of THF GPs

**Fig. 4:**
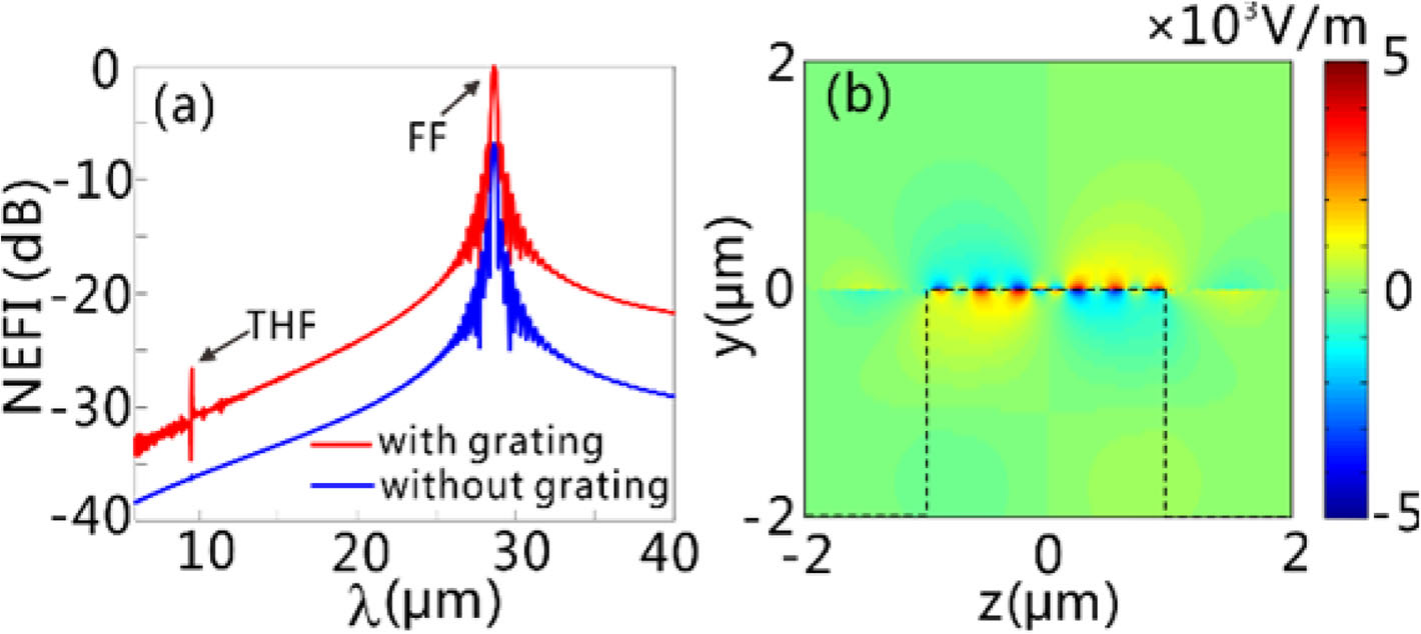
Generation of THF GPs on the GSSDG. **a** The NEFI for the structure with (red line) and without (blue line) grating normally illuminated by CW light with the power density of 0.11 MW/cm^2^ and the central wavelength of 28.62 μm. The two peaks in the red line denote the FF GPs (*λ* = 28.62 μm) and the generated THF GPs (*λ* = 9.54 μm), respectively. **b** The distribution of the real part of *E*_*y*_ for the generated THF GPs. The black dashed line in **b** represents the outlines of the silicon layer. The structural parameters of GSSDG are the same as those in Fig. 3

We then compare the electric field intensity of the THF GPs on the graphene surface when the graphene sheets are sustained with and without grating. The boundary conditions in FDTD simulations are the same as those used in Figs. 2 and 3. The normalized electric field intensity (NEFI) as a function of wavelength is presented in Fig. 4a, when the graphene sheets are illuminated by normal-incidence continuous-wave (CW) light with the power density of 0.11 MW/cm^2^ and the central wavelength of 28.62 μm. Here, the NEFI is obtained by normalizing the electric field intensity to its value at 28.62 μm (FF) with the grating structure. It can be observed that, an apparent peak occurs at THF in the NEFI spectrum with grating structure (GSSDG), compared to the NEFI spectrum without grating involved. Defining the CE as ${\int}_0^p{P}_y^{THF} dz/\left({P}^{FF}p\right)$, where ${P}_y^{THF}$ is the *y* component of poynting vector at THF, and *P*^*FF*^ is the power density of the incident light, the CE reaches as high as 5.71 × 10^−7^ for the GSSDG. It can be easily inferred that, the excitation of the FF GPs contributes to the enhancement of the CE of THF GPs. The field distributions of real part of *E*_*y*_ at THF shown in Fig. 4b validate the generation of THF GPs on the graphene surface.

### The GSSDG as a Light Source for Mid-infrared Silicon Photonics

**Fig. 5:**
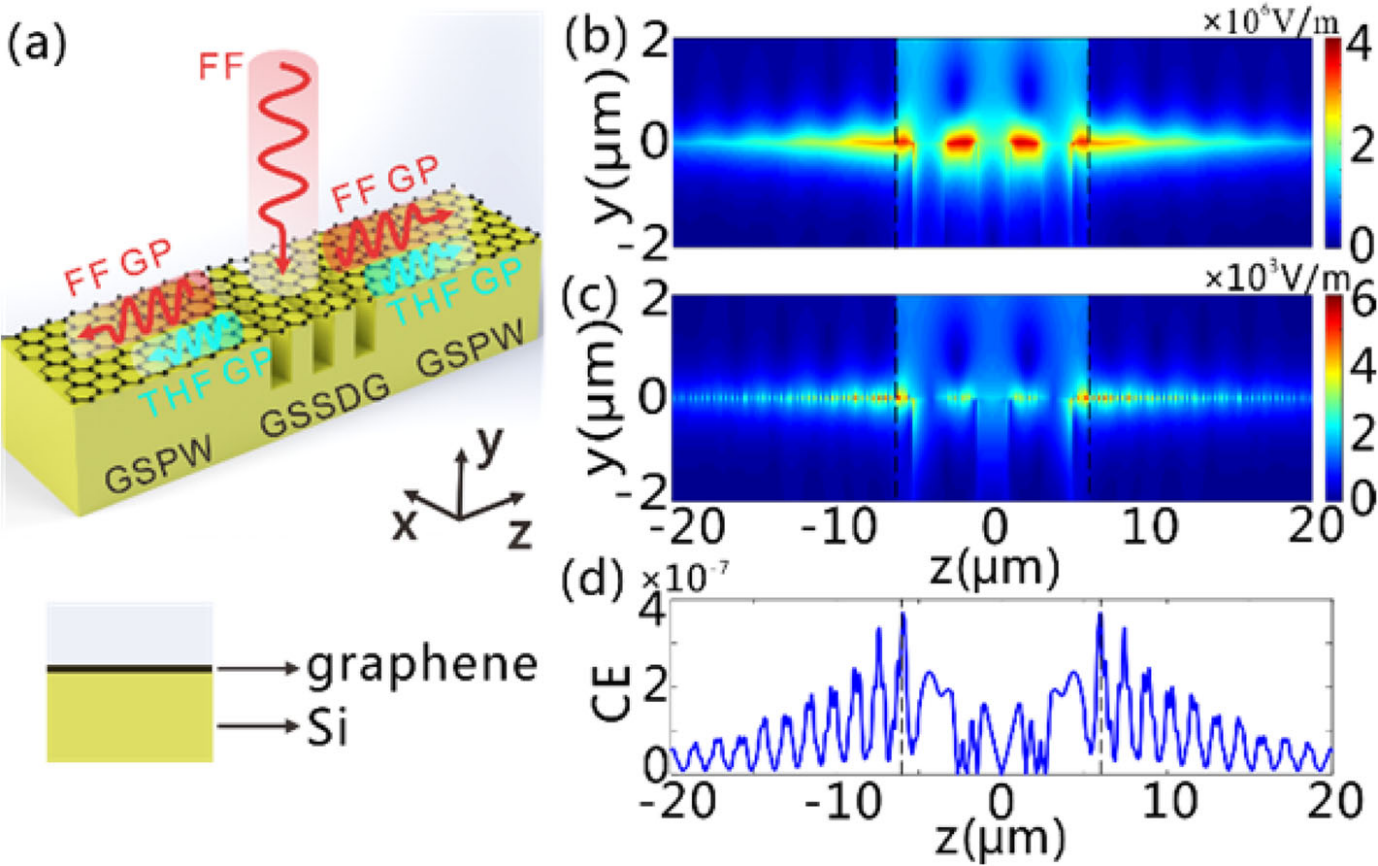
Generation of FF and THF GPs on the GSSDG and the connected GSPWs. **a** The schematics of the GSSDG and the connected GSPWs on both sides when a normal-incidence x-polarized plane wave of FF illuminates the structure. The THF GPs are generated and guided along the two GSPWs after the FF GPs are excited in the GSSDG. The cross section of the GSPW is presented in the panel below, in which the graphene sheets and Si layer are denoted. **b**, **c** The |*E*| distributions of **b** FF and **c** THF GPs in the *y*-*z* plane as the grating section is illuminated by CW light with the power density of 0.19 MW/cm^2^ at 28.62 μm. **d** The CE of THG along the *z* direction. The black dashed lines in **b**–**d** represent the interfaces between the GSSDG and GSPWs. In **b**–**d**, *N*_*p*_ is set at three

We next consider the use of the GSSDG plasmonic wavelength converter to directly provide light source for the silicon integrated photonic circuits. As an example shown in Fig. 5a, two graphene-silicon plasmon waveguides (GSPWs) are attached to the GSSDG on both sides. The GSPWs are chosen such that they are capable of guiding GPs over a wide spectral band covering the FF and THF GPs. Since the modal field distributions of the FF and THF GP modes in the GSSDG (asymmetrical respect to graphene surface) present strong similarity with the GP modes supported on the GSPW, one can thus deduce that, once the grating section is illuminated with normal-incidence FF waves, the generated FF and THF GPs above the grating region can be efficiently coupled to the GSPWs on both sides. We performed FDTD simulations to validate our prediction. The prefect matched layer boundaries are used in both *y* and *z* directions in the modeling. We simulated a normal-incidence FF light wave that impinges the grating section, and showed the electric field distributions for the FF and THF GPs (Fig. 5b, c). A total-field/scattered-field light source is used to ensure that only the grating section is illuminated with incident light in the simulation [33]. Perfectly matched absorbing boundary was used to totally absorb all the light waves that reach the boundary of the computation region. Figure 5b shows the FF GPs are excited on the graphene surface above the grating and then propagate along the GSPWs on both sides. From Fig. 5c, we can further find the appearance of the THF GPs on the graphene surface, both in the grating section and in the GSPWs. Here, the CE is defined as

8 $$ \mathrm{CE}=\int {P}_z^{T\mathrm{HF}}\mathrm{dz}/\left({P}^{\mathrm{FF}}{N}_pp\right) $$

where ${P}_z^{\mathrm{THF}}$ is the *z*-component of poynting vector at THF, $\int {P}_z^{\mathrm{THF}}\mathrm{dz}$ is the output power density of THF GP in the GSPW, *P*^FF^ is the power density of the incident FF light waves, and *N*_*p*_ is the number of grating period. One can see from Fig. 5d that, the CE of THG reaches the maximum value of 3.68 × 10^−7^ (− 64.3 dB) at the grating boundary and exponentially attenuates along the propagation direction due to the ohmic absorption loss from graphene.

**Fig. 6:**
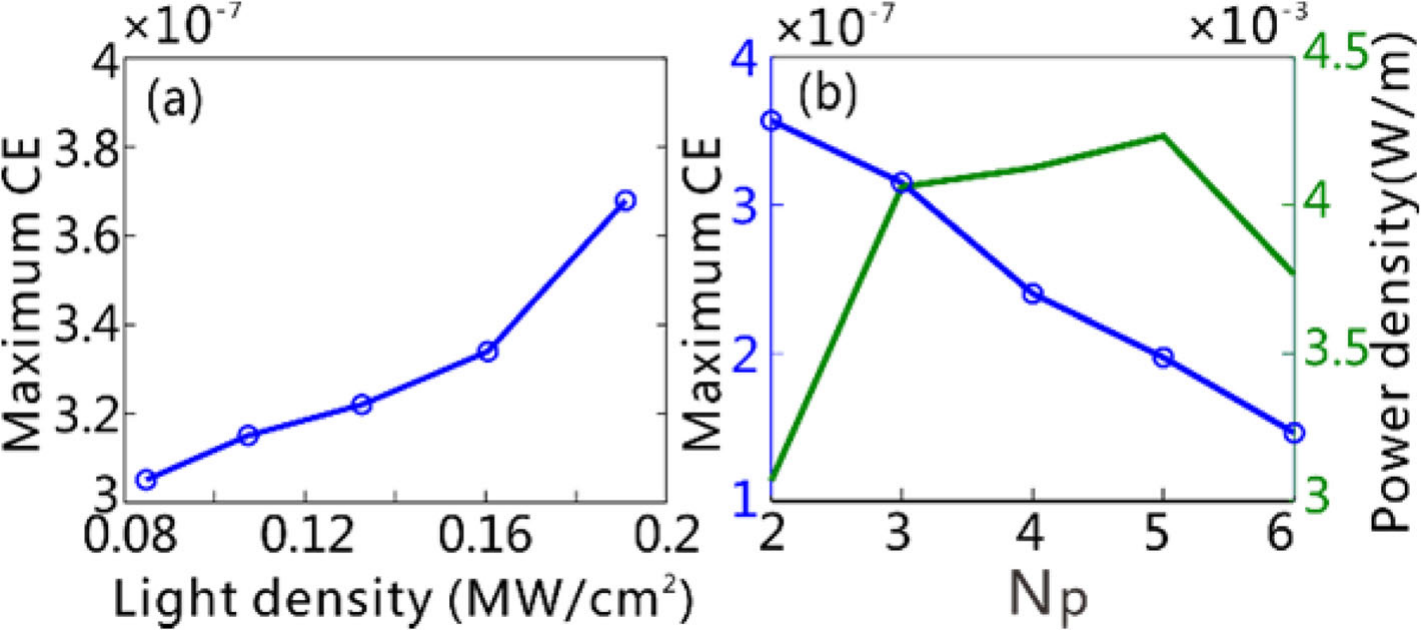
**a** The maximum CE of THG as a function of the incident power density for *N*_*p*_ = 3. **b** The maximum CE and the maximum output power density of THG as a function of the number of grating periods *N*_*p*_ used, respectively. The incident power density is fixed at 0.11 MW/cm^2^

It is important to discuss the factors that affect the THG CE, which is key to evaluate the device performance of a THG wavelength converter. For a THG process, one always expects to achieve the largest CE with a relatively small pump power. Previous studies demonstrated that, increasing the local field intensity in the third-order nonlinear materials yields a remarkable improvement of the CE of THG with a significantly reduced pump power [3, 4, 7]. Figure 6a shows the influence of the power density of incident light waves on the maximum CE in the GSPWs, which is increased with the power density. Note that the maximum CE reaches up to 3.68 × 10^−7^ even if the power density of incident light waves is as low as 0.19 MW/cm^2^, which is 6–7 orders of magnitude smaller than those within the same spectral band [22, 23]. We show in Fig. 6b that the used number of grating period, *N*_*p*_, affects the CE in the GSPWs as well. When *N*_*p*_ is increased, a reducing portion of the THF GPs generated in the center of the grating reaches the GSPWs because of the enhanced propagation loss induced by graphene absorption. Nevertheless, the input power, associated with *N*_*p*_, presents linear enhancement. Therefore, the maximum CE of the THF GPs decreases with the increased *N*_*p*_. We emphasize here that the absolute output power density of THF GPs should be more meaningful to guide the design of a THG wavelength converter for practical applications, once the incident power density is fixed. Although the maximum CE of the THF GPs lies at *N*_*p*_ = 2 in our case, the output power density of THG approximates the maximum when *N*_*p*_ ≥ 3 (Fig. 6b). Therefore, we have employed 3 periods of grating for the demonstration of the generation of THF GPs in the GSPWs. For future experimental implementation with the current design, the area of input FF source exceeds the grating region and is kept constant when generating THF GP with different grating numbers. In this case, the CE should be written as

9 $$ \mathrm{CE}=\int {P}_z^{\mathrm{THF}}\mathrm{dz}/\left({P}^{\mathrm{FF}}S\right) $$

where the area of the FF source, *S*, is constant. Thus, the output power density will be proportional to the CE, and hence, one must properly select the optimal grating number to maximize the output power density of THF GP.

The physical characteristics of graphene may also affect the device performance of THF GPs once the studied structure shown in Fig. 5a is ready. The Fermi energy, *μ*_*c*_, and the number of graphene layers will significantly change the resonance wavelength of FF GPs (Fig. 2f, i) and therefore affect the generation wavelength of THF GPs as well. In contrast, the *τ*, associated with the carrier mobility *μ*, barely influences the resonance wavelength of FF GPs as well as the generation wavelength of THF GPs (Fig. 2c). However, the propagation loss of FF GPs and THF GPs can be reduced by the use of a much larger *τ* (Fig. 2b), which thus notably increases the CE of THF GPs. Considering that a carrier mobility of *μ* > 10 m^2^V^−1^s^−1^ (*τ* > 1.5 ps) is achievable in the experiment [32], our simulation results (*τ*= 0.5 ps) shown in (Figs. 3, 4, 5 and 6) can conservatively present the device performance of the THF GP generator in Fig. 5a.

Finally, it is worth discussing the influences of surface roughness of graphene sheets on the device performance. The surface roughness of graphene could potentially scatter plasmon, and hence, the plasmon loss will be enhanced [34]. The proposed THF GP generator shown in Fig. 5 can be fabricated based on the current micro/nano fabrication technology. One can first spin a 270-nm-thick polymethyl methacrylate (PMMA) onto the silicon substrate. The PMMA layer is developed with MIKE\IPA after a subsequent electron-beam lithography process. After that, a 60-nm-thick Cr layer is deposited on the resist with electron-beam evaporation deposition method. The silicon grating substrate can be formed with etching techniques such as inductively coupled plasma machine. Followed by wet etching method, the residual Cr layer is removed by wet etching method. At last, the graphene sheets are transferred onto the silicon grating to form the final structure shown in Fig. 5.

## Conclusion

We have numerically demonstrated the generation of the THF GPs in a graphene sheet on silicon gratings with the normal-incidence plane waves in mid- and far-IR regions. It was shown that THF GPs are generated and transmitted on the graphene surface, and the CE is dramatically enhanced due to the significantly increased field intensity of the excited FF GPs in combination of the large third-order nonlinear susceptibility of graphene. The generated THF GPs can be conveniently coupled to a GSPW, which greatly facilitates the integration of the graphene-based wavelength converter on a silicon platform. Our proposal can stimulate making graphene-based light sources for mid- and far-infrared photonics on a silicon platform and hence broaden the functionalities of silicon photonics, such as signal processing, spectroscopy, and sensing.

## Abbreviations

CE: Conversion efficiency
CW: Continuous wave
FDTD: Finite difference time domain
FF: Fundamental frequency
GP: Graphene plasmon
GSPW: Graphene-silicon plasmon waveguide
GSSDG: Graphene sheets sustained by dielectric grating
PMMA: Polymethyl methacrylate
THF: Third-harmonic frequency
THG: Third-harmonic generation
